# FOXH1 promotes lung cancer progression by activating the Wnt/β-catenin signaling pathway

**DOI:** 10.1186/s12935-021-01995-9

**Published:** 2021-06-05

**Authors:** Jun Zhang, Xian Zhang, Shasha Yang, Yanqiu Bao, Dongyuan Xu, Lan Liu

**Affiliations:** 1 Department of Morphological Experiment Center, Medical College of Yanbian University, Yanji, Jilin 133000 China; 2 Department of Histology and Embryology, Jilin Medical University, Jilin, Jilin 132013 China; 3 Department of General Surgery, Affiliated Hospital of Yanbian University, Yanji, Jilin 133000 China; 4 Department of Pathology, Affiliated Hospital of Yanbian University, Yanji, Jilin 133000 China

**Keywords:** FOXH1, Lung cancer, Proliferation, EMT, β-catenin

## Abstract

**Background:**

The expression of forkhead box protein H1 (FOXH1) is frequently upregulated in various cancers. However, the molecular mechanisms underlying the association between FOXH1 expression and lung cancer progression still remain poorly understood. Thus, the main objective of this study is to explore the role of FOXH1 in lung cancer.

**Methods:**

The Cancer Genome Atlas dataset was used to investigate FOXH1 expression in lung cancer tissues, and the Kaplan–Meier plotter dataset was used to determine the role of FOXH1 in patient prognosis. A549 and PC9 cells were transfected with short hairpin RNA targeting FOXH1 mRNA. The Cell Counting Kit-8, colony formation, soft agar, wound healing, transwell invasion and flow cytometry assays were performed to evaluate proliferation, migration and invasion of lung cancer cells. Tumorigenicity was examined in a BALB/c nude mice model. Western blot analysis was performed to assess the molecular mechanisms, and β-catenin activity was measured by a luciferase reporter system assay.

**Results:**

Higher expression level of FOXH1 was observed in tumor tissue than in normal tissue, and this was associated with poor overall survival. Knockdown of FOXH1 significantly inhibited lung cancer cell proliferation, migration, invasion, and cycle. In addition, the mouse xenograft model showed that knockdown of FOXH1 suppressed tumor growth in vivo. Further experiments revealed that FOXH1 depletion inhibited the epithelial-mesenchymal transition of lung cancer cells by downregulating the expression of mesenchymal markers (Snail, Slug, matrix metalloproteinase-2, N-cadherin, and Vimentin) and upregulating the expression of an epithelial marker (E-cadherin). Moreover, knockdown of FOXH1 significantly downregulated the activity of β-catenin and its downstream targets, p-GSK-3β and cyclin D1.

**Conclusion:**

FOXH1 exerts oncogenic functions in lung cancer through regulation of the Wnt/β-catenin signaling pathway. FOXH1 might be a potential therapeutic target for patients with certain types of lung cancer.
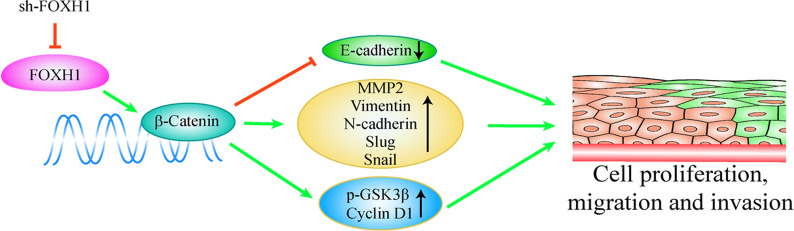

**Supplementary Information:**

The online version contains supplementary material available at 10.1186/s12935-021-01995-9.

## Background

Lung cancer is the second most common cancer type worldwide in both men and women. It poses a considerable threat to human health and life and has been the leading cause of cancer death so far, accounting for about 25% of all cancer-related deaths [1, 2]. Compared to other malignant tumor types, lung cancer has higher incidence and mortality rates, which is attributed to delayed diagnosis [3]. In advanced lung cancer, the acquired ability of the cancer cells to move and invade nearby tissues is associated with their high metastatic potential. Numerous regulatory mechanisms, such as epithelial-mesenchymal transition (EMT), are involved in tumor metastasis. EMT is an important physiological process that allows epithelial cells to gain the invasive activity and motility of mesenchymal cells [4]. The int/Wingless family (Wnt)/β-catenin signaling pathway regulates cell morphogenesis, gene transcription, differentiation, and proliferation [5, 6]. Slug, Vimentin, matrix metalloproteinases (MMPs), and other downstream target genes of Wnt/β-catenin are key regulators of EMT [7]. In the early stages of metastasis, cancer cells undergo an EMT-like process controlled by active Wnt/β-catenin signaling [8]. Therefore, elucidation of the mechanisms underlying tumor invasion and metastasis may be the key to improving survival rates of patients with lung cancer.

Forkhead box protein H1 (FOXH1), also known as Fast1, is a member of the FOX family, an evolutionarily conserved transcription factor family [9]. FOXH1 has been studied in several types of cancer cells. Liu et al. confirmed that FOXH1 is overexpressed in breast cancer cells, promoting their proliferation and invasion [10]. In addition, FOXH1 has been reported to be tightly associated with colorectal cancer progression and could serve as an independent prognostic factor. FOXH1 overexpression promotes the proliferation, migration, and invasion of colorectal cancer cells in vivo by down-regulating E-cadherin level [11]. In acute myeloid leukemia, FOXH1 is a critical mediator of the functions of mutant p53 that binds to and regulates stem cell-associated genes and transcriptional programs. Studies have shown that mutant p53 appears to promote leukemia by enforcing the expression of FOXH1, which is involved in promoting stemness and cell plasticity during hematopoiesis. Furthermore, FOXH1 binds to Smad2/3 and mediates transforming growth factor beta (TGF-β) signaling, which is associated with tumor development and progression [12, 13]. FOXH1 has also been linked with the EMT process in *Xenopus tropicalis*. EMT is an evolutionarily conserved process that is considered essential for normal embryonic development. Recent evidence has, however, indicated that EMT is also implicated in the processes of cancer progression and metastasis [14–16]. However, the biological functions of FOXH1 in lung cancer progression still remains poorly understood. Therefore, the main objective of this study was to explore the role of FOXH1 in lung cancer.

In this study, we found that FOXH1 expression was significantly upregulated in lung cancer tissues and closely related to patient prognosis. Knockdown of FOXH1 significantly suppressed the proliferation, migration and invasion of lung cancer cells. Animal experiments showed that FOXH1 facilitated tumorigenesis of lung cancer in vivo. Further investigation into the mechanism underlying this effect proved that Wnt/β‐catenin signaling pathway was activated by FOXH1 to promote the lung cancer progression. Our study indicated that targeting FOXH1 might be beneficial to some patients with lung cancer.

## Materials and methods

### Antibodies and reagents

Primary antibodies against FOXH1 (Fast1/2), MMP2, Vimentin, N-cadherin, Snail, E-cadherin, Slug, β-actin, β-catenin, p-GSK-3β and cyclinD1 (Santa Cruz Biotechnology, Santa Cruz, CA, USA), HRP-conjugated anti-mouse, anti-goat, anti-rabbit secondary antibodies (Zhongshan golden bridge biotechnology, BJ, China), Giemsa stain (Solarbio, BJ, China), Soft agar (Becton Dickinson and Company, Bedford, MA, USA), CCK-8 reagent (BestBio, Shanghai, China) were used in this study.

### Cell culture

The human lung cancer cell lines, i.e. PC9, A549, H1299 (NCI-H1299, carcinoma, non-small cell lung cancer), H460 (NCI-H460, carcinoma, large cell lung cancer) and H1975 (NCI-H1975, adenocarcinoma, non-small cell lung cancer), together with human bronchial epithelial cell line (16HBE) were provided by the American Type Culture Collection (ATCC; Manassas, VA, USA). All cell lines were maintained and cultured in Dulbecco's modified Eagle's medium (DMEM Gibco, NY, USA) supplemented with 10% Penicillin–Streptomycin Liquid (Solarbio, BJ, China), 10% fetal bovine serum (FBS, Gibco, NY, USA) in an incubator containing 5% CO_2_ maintained at 37℃.

### Gene coexpression with FOXH1 in TCGA dataset

The Cancer Genome Atlas (TCGA) is a large-scale cancer genomics program that has molecularly characterized 33 primary cancer types including 3 subtypes of lung cancer. The Oncomine database is a database based on a gene chips and a integrated platform of data mining. In this data, the conditions of screening and mining data can be set up according to own requirements. In this study, the screening conditions were set as: (1) “Cancer Type: Lung cancer”; (2) “Gene: FOXH1”; (3) Dataset Name: TCGA Lung 2; (4) “Analysis Type: Cancer vs Normal Analysis”; (5) Threshold setting conditions (*P* value < 0.05). As a result, the gene expression profiles including “Lung Adenocarcinoma”, “Papillary Lung Adenocarcinomar” and “Squamous Cell Lung Carcinoma” were selected from the Oncomine database using the above filters. A total of 1016 specimens including 397 normal lung tissues and 619 lung cancer tissues were available in this study (Data reporter ID: 08–145,671,225; RefSeq Genes: UCSC refGene, July 2009, hg18, NCBI 36.1, March 2006).

### Western blotting analysis

For preparing entire cell lysates, cells underwent lysing treatment on ice in RIPA lysis buffer that contained 150 mM NaCl, 50 mM Tris–HCL [pH 7.4], sodium deoxycholate (0.25%), NP40 (1%), 1 mM EDTA, sodium dodecyl sulfate [SDS, 0.1%], 1 mM PMSF. 10 μg/ml aprotinin and 10 μg/ml leupeptin. SDS-PAGE was used to resolve 20 μg of the protein of the cell or the tissue lysate, followed by a transfer to a nitrocellulose membrane (Millipore, Billerica. MA. USA). Tris-buffered saline solution that contained 0.05% Tween 20 were used for blocking the resultant for 1 h at room temperature. The relevant antibodies were added to probe the blots overnight at 4℃. Followed by washing, the blots were probed again with the species-specific secondary antibody coupled to the horseradish peroxidase. Dilutions of all primary and secondary antibodies are listed in Additional file 1: Table S1. An ECL prime Western blotting kit (Amersham Pharmacia Biotech, Buckinghamshire, UK) were used to assess the immunoreactivity, following the instruction of manufacturer.

### Lentiviral vectors and transduction expressed by short hairpin RNA (shRNA)

Lentivirus vectors which encode a shRNA targeting FOXH1 (target sequence: CCGGAGTGAGGGCTTCAGCATCAAGCTCGAGCTTGATGCTGAAGCCCTCACTTTTTTTG; Product Type: SHCLND-NM_003923; TRC number: TRCN0000431226) or shRNA non-targeting control (target sequence: CCGGCAACAAGATGAAGAGCACCAACTCGAGTTGGTGCTCTTCATCTTGTTGTTTT; Product Type: SHC202V) were used to transduce A549 and PC9 cells following the instruction of manufacturer (Sigma Chemical Co). Briefly, 3 × 10^5^ cells were cultured overnight in a 6-well plate, followed by a transduction with lentiviral particles at 1 multiplicity of infection (MOI) with 8 µg/ml polybrene. The transduction efficiency of each vector was confirmed via the western blotting analysis.

### CCK8 assay

The proliferation of cell was measured using the Cell Counting Kit 8 (CCK-8) assay. Cells receiving various treatment or transfection (1 × 10^3^ cells/well) were planted in the 96-well plates. Then 10 µl CCK-8 solution was used to mix cells in every well respectively after being incubated for 0, 24, 48, 72, 96 and 120 h. Another 4 h of incubation helped to obtain the absorbance values of 450 nm wavelength.

### Colony formation assay

Cell culture medium was used to adjust the concentration of the cells to 2 × 10^3^ cells/ml. Then 2 ml cells were plated in each of the 6-well plates, followed by 14 days of incubation. When colonies appeared, discard the supernatant. The cells were washed twice with phosphate-buffered saline (PBS), fixed with 4% paraformaldehyde for 15 min, and stained with Giemsa solution for 30 min. Finally, colony formation was assessed.

### Soft agar colony formation assay

Cells were suspended in the growth medium that contained 0.35% agar and 1.5 × 10^5^ cells were plated in 6-cm plates (3 ml/well) on the top of a layer of growth medium that contained 0.7% agar (5 ml/well). The agar was added with 500 µl of growth medium coupled with FBS (10%). The cells were incubated for two weeks at 37℃, then the formation of viable colonies was observed using an optical microscope.

### Flow cytometry

After the cultured cells were trypsinized and washed, 75% ethanol were added to suspend the cells and keep at 4℃ overnight to fix the cells. The fixed cells underwent 30 min of incubation with the RNase reagent at 37℃, followed by 30 min of staining with the propidium iodide (PI; 1:100, Propidium iodide cycle Detection kit II, BD Bioscience, USA). The stained cells were acquired using FACS Caliber (BD Company, USA) and analyzed with the standard software. The data were presented as percentages of cells distributed at cell cycle in G1 phase, S phase, and G2 phase, respectively. At least three parallel tests were conducted.

### Animal experiments

The present experiment employed female BALB/c nude mice were purchased from Charles River Laboratories (Charles river, Beijing, China), aged at four weeks and maintained under specific pathogen free (SPF) condition in the animal care facility. The mice' health is monitored twice a day during feeding and no adv. adverse events were observed the environmental conditions were 21 ± 2 ℃ and 55% ± 10% humidity. Each mouse was placed in a 300 × 210 × 130 mm size cage, they drank water freely and were provided with enough food. All the mice were anesthetized with isoflurane that controlled by a small animal anesthesia machine. The transduction of A549 cells relied on a lentivirus vector that encoded an shRNA targeting FOXH1. After cell harvest, we resuspended 3 × 10^6^ cells in 60 μl DMEM coupled with 20 μl Matrigel (Becton Dickinson and Company, Bedford, MA, USA), followed by orthotopical injection into mouse’ right shoulder. Tumor diameter was recorded using a vernier caliper every 2 days. Tumor volume was calculated follows formula: tumor volume (mm^3^) = (length × width^2^)/2. Tumor nodule collection was performed after the euthanization of mouse on day 36. According to the AVMA Guidelines for the Euthanasia of Animals, all the mice were euthanized with an intraperitoneal injection of a three-fold dose of barbiturates. After that we removed tumors immediately and measured the length, width and weight of the tumors. No mice died accidentally during feeding. All surgical procedures and experimental protocols were approved by the Animal Care and Use Committee of Yanbian University Faculty of Medicine. The procedures were performed according to the recommendations of the Yanbian University Institutional Animal Care Use Committee (IACUC).

### Transwell assay

The transfected A549 and PC9 cells were resuspended in 100 μl DMEM free of serum and plated in upper chamber of transwell device, with 5 × 10^5^ cells/well. 600 μl complete medium were added into lower chamber as the chemical attractant. After 48 h of incubation in the incubator at 37 °C, the cells in upper chamber were removed, and 4% paraformaldehyde were used to fix the cells that invaded the lower chamber, followed by washing in PBS twice and staining with Giemsa solution.

### Wound healing assay

The transfected A549 and PC9 cells were firstly seeded into a 6-well plate at 3 × 10^5^ cells/well. After the cells reached 80% confluence, the tip of a 200 μl pipette was used to scratch the monolayers, and the culture was continued in the FBS-free medium. Subsequently, the wound healing occurred under an inverted microscope and photographed at 24 and 72 h using a digital camera (Olympus, Japan). Image J was used to analyze the results of the scratch experiment and calculate the scratch area.

### Luciferase reporter gene assay

Luciferase Assay System (Promega, Madison, WI, USA) was used to measure the transcriptional activity assays following the instruction of manufacturer. TOP flash/FOP flash reporter plasmids were used to assess the transcriptional activity dependent on TCF/LEF in Transfected sh-FOXH1 A549 cells and PC9 cells. A Dual-Luciferase Reporter (DLR) Assay Kit (Promega) was applied to measure the luciferase activity following the manufacturer’s instruction. A luminometer (Lumat LB9507, Berthold, Bad Wildbad, Germany) was employed to gauge the Firefly and Renilla luciferase activity for the convenience of normalization.

### Immunohistochemistry (IHC)

Formalin-fixed, paraffin-embedded tissue sections were cut to 4 μm thickness. After rehydration, the slides were pretreated and deparaffinized through microwave epitope retrieval process (750 W for 15 min in 10 mM citrate buffer, pH 6.0) before application of the primary antibody. The primary antibody (FOXH1 1:100, Ki-67 1:100) was detected using a secondary biotinylated antibody and development by DAB substrate (DAKO, Glostrup, Denmark). Dilutions of primary antibodies are listed in Additional file 1 Table. Staining showed absolute specificity to the nucleus without discernible off-target signal [17]. Counter-staining was completed with Meyer’s hematoxylin.

### Gelatin zymography for assaying MMP2

MMP2 activity was assayed by gelatin zymography. The culture supernatants (serum free) from both PC9 and A549 untreated control cells, sh-NC transfected cells and sh-FOXH1 transfected cells were collected after 48 h. The supernatants were centrifuged at 3000 rpm for 10 min. The proteins in the samples were separated by SDS-PAGE. The SDS-PAGE gel was preceded further with the buffers according to Nanjing Xinfan Biology’s protocol (http://www.njxfbio.com/Products-19186253.html). Components of MMP zymography analysis kit are listed in Additional file 2: Table S2. After electrophoresis, the gels were washed with 2.5% Triton-X100 in Tris–HCl (pH 7.5), 5 mM NaCl to remove SDS, incubated in 50 mM Tris–HCl (pH 7.5), 10 mM CaCl_2_ for 24 h at 37 °C, and stained with Coomassie Brilliant Blue [18, 19].The intensity of the bands from both the control and the treated samples were analyzed by ImageJ Software.

### Analysis

SPSS 17.0 software (IBM, USA) and SigmaPlot 14.0 software (Systat Software, USA) were applied to the statistical analyses. All the experiments were carried out repeatedly for three times, with data represented as the mean ± SD. Student's t-test was used to compare continuous quantities in different groups, and one-way analysis of variance (ANOVA) was carried out for comparisons among  ≥ 2 groups. Kaplan Mier methods and logrank test were used to analyze survival data. The *p* value < 0.05 was considered with statistical significance.

## Results

### FOXH1 expression is upregulated in lung cancer

**Fig. 1:**
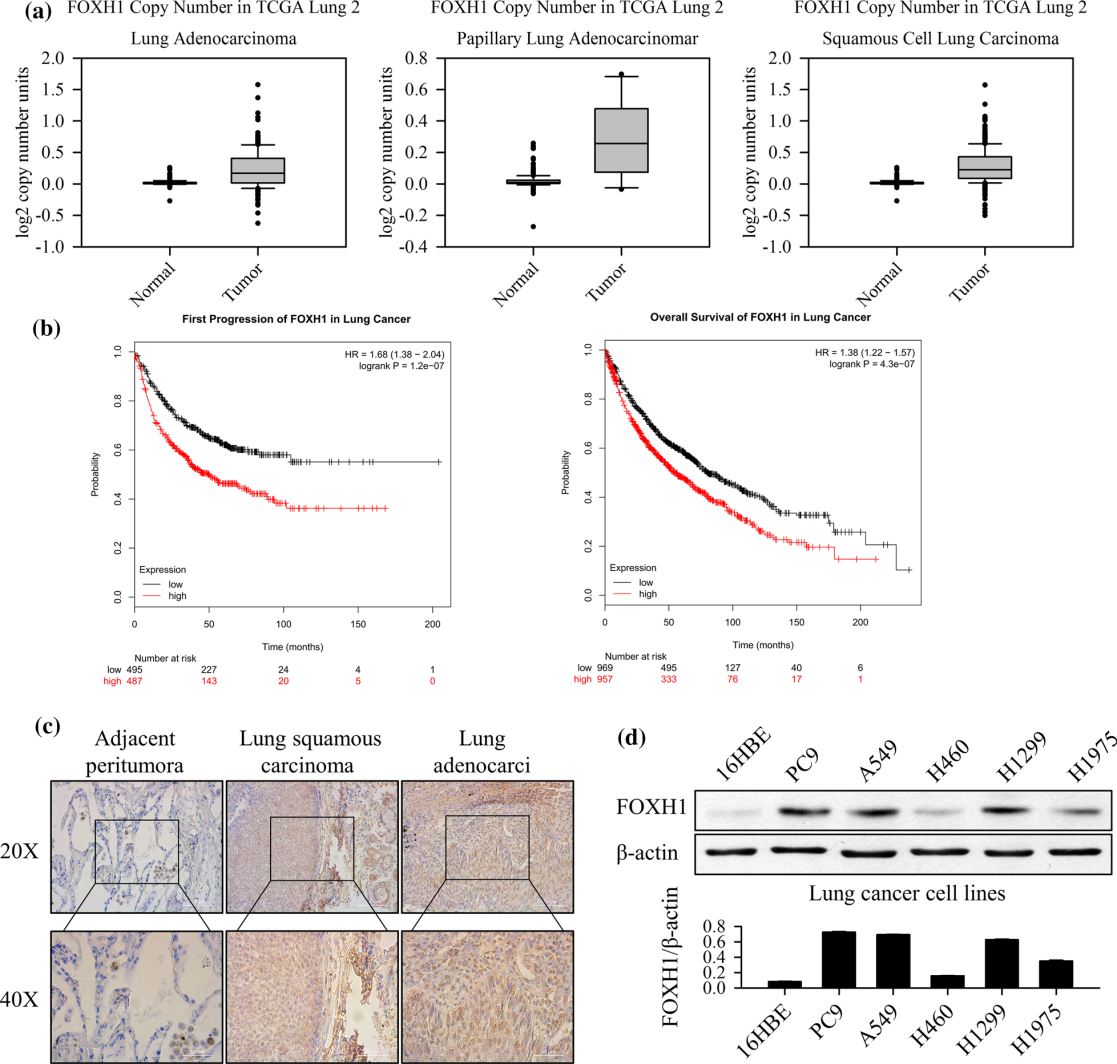
The expression of FOXH1 in lung cancer tissues and cell lines. **a **The lung cancer tumor exhibited an obviously higher FOXH1 expression than normal group in the TCGA database. **b** Higher expression of FOXH1 in lung cancer causes lower survival time of lung cancer. **c** IHC staining for FOXH1 in lung cancer tissues, negative expression of FOXH1 in para-carcinoma tissues, strong expression of FOXH1 in tumor tissues was mainly located in the nucleus. **d** Western blot analysis of FOXH1 expression in the lung cancer cell lines (PC9, A549, H460, H1299 and H1975) and human bronchial epithelial cells (16HBE)

The Cancer Genome Atlas (TCGA) is a large-scale cancer genomics program that has molecularly characterized 33 primary cancer types including 3 subtypes of lung cancer. In order to test whether FOXH1 is differentially expressed in lung cancer samples, the normalized gene expression data from TCGA were extracted, and FOXH1 gene expression levels in lung cancer samples were compared with normal tissues using t-tests. The results showed that FOXH1 expression levels are significantly higher in lung adenocarcinoma (*p* < 0.05), papillary lung carcinoma (*p* < 0.05) and squamous cell lung carcinoma (*p* < 0.05) compare to normal samples. The mean fold change of FOXH1 expression in lung adenocarcinoma, papillary lung adenocarcinoma and squamous cell lung carcinoma were 0.168, 0.258, and 0.225, respectively (Fig. 1a). Next, to determine whether FOXH1 is a prognostic marker for lung cancer, we included lung adenocarcinoma samples with patient’s cancer progression and survival data available in TCGA dataset. The lung cancer patients were stratified into FOXH1 high and FOXH1 low groups using the median of FOXH1 gene expression levels in lung cancer samples. Both progression free survival and overall survival were assessed by Kaplan–Meier plots and logrank tests (Fig. 1b). Patients whose samples have low FOXH1 expression level have significantly longer progression free survival and overall survival. Poorer prognosis observed for lung adenocarcinoma patients with higher FOXH1 expression in tumor implies FOXH1 is a prognosis marker and can be a potential treatment target for lung cancer.

The immunohistochemistry staining was applied to determined FOXH1 protein expression levels in the paraffin-embedded lung cancer tissues. Representatively, immunohistochemical detection of FOXH1 in the lung cancer and normal lung tissues according to different samples of lung cancer revealed that the immunoreactivity of FOXH1 was barely expressed in surrounding non-tumor and normal lung tissue compared with the tumor. FOXH1 protein expression was highly detected in the nuclei of tumors (Fig. 1c). To establish the functional role of FOXH1 in lung cancer, its expression levels were examined in various lung cancer cell lines and the 16HBE cell line derived from normal lung epithelial cells. As shown in Fig. 1d, the expression of FOXH1 in all the lung cancer cell lines was higher than that in 16HBE normal cell line. While A549, PC-9 and H1299 cells had significantly higher FOXH1 expression, the FOXH1 expression in H460 and H1975 cells were only marginally higher. Since A549 and PC9 cell lines simulate lung cancer tissue in terms of the FOXH1 expression level, they were selected for the further experiments.

### Knockdown of FOXH1 suppresses growth of lung cancer cells

**Fig. 2:**
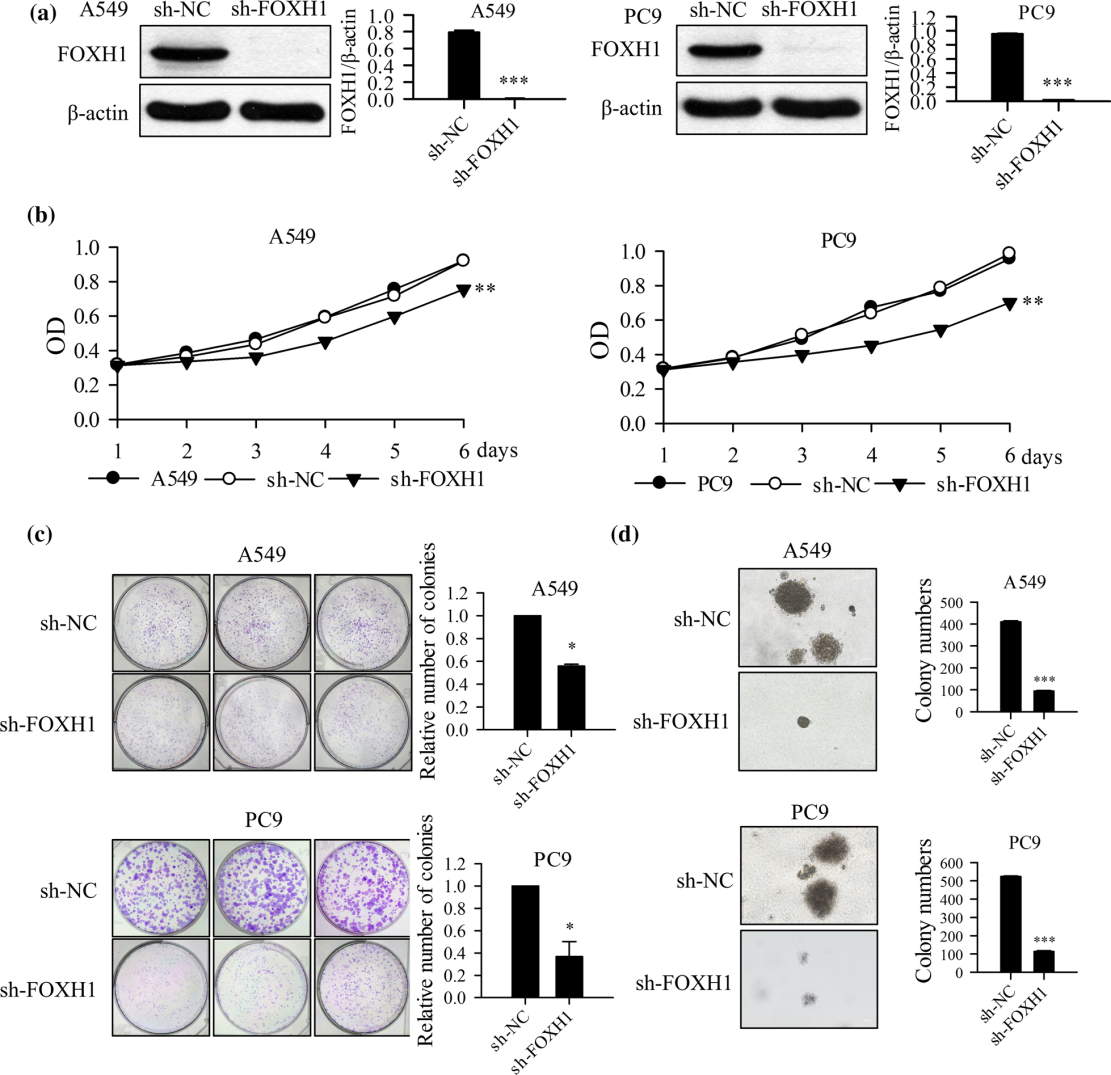
Knockdown of FOXH1 suppresses proliferation of the lung cancer cells. **a** A549 and PC9 cells transfected with the sh-FOXH1 plasmid and the sh-NC (sh-Nontarget Control) plasmid, FOXH1 expression was examined by western blot (***p < 0.001, vs. sh-NC). **b** CCK8 assay applied to determine the cell viability in sh-FOXH1 transfected A549 and PC9 cells (**p< 0.01 vs. sh-NC). **c** Cell colony formation assay was used to determine colony forming capability in sh-FOXH1 transfected A549 and PC9 cells. The relative number of colonies is performed with histogram. (*p< 0.05 vs. sh-NC) **d** Cellular proliferation in sh-FOXH1 transfected A549 and PC9 cells were evaluated via the soft agar colony formation assay. The colonies were counted (***p< 0.001 vs. sh-NC)

To test the hypothesis that FOXH1 is an oncogene whose elevated expression in lung cancer cells drives the tumor growth and proliferation, effects of FOXH1 knockdown on cellular growth and cell cycles of lung cancer cells were examined in subsequent experiments. The two lung cancer cell lines, PC9 and A549, were transfected with either a sh-RNA specifically targeting FOXH1 (sh-FOXH1) or a negative control sh-RNA (sh-NC), then a Western blot assay was applied. As shown in Fig. 2a, FOXH1 was effectively knocked down in both sh-FOXH1 transfected lung cancer cell lines and its expression level reduced > 95% 72 h after transfection compared to cells transfected with negative control sh-RNA (sh-NC). And the FOXH1 expression level stayed low in the following 14 days. CCK8 assays revealed that the proliferation of lung cancer cells was suppressed by FOXH1 silencing while the negative control cells proliferated at the same speed as the un-transfected cells (Fig. 2b). Cell colony formation and soft agar colony formation assays were used to verify the effect of FOXH1 knockdown on cell proliferation in vitro. The colony forming capability possessed by A549 and PC9 cells was markedly decreased upon FOXH1 depletion (Fig. 2c) and the soft agar colony formation assay showed a significant decrease in colony counts when the A549 and PC9 cells were depleted of FOXH1 (Fig. 2d). These results indicated that knockdown of FOXH1suppresses growth of lung cancer cells.

### FOXH1 down-regulation inhibits lung cancer cell proliferation in vivo

**Fig. 3:**
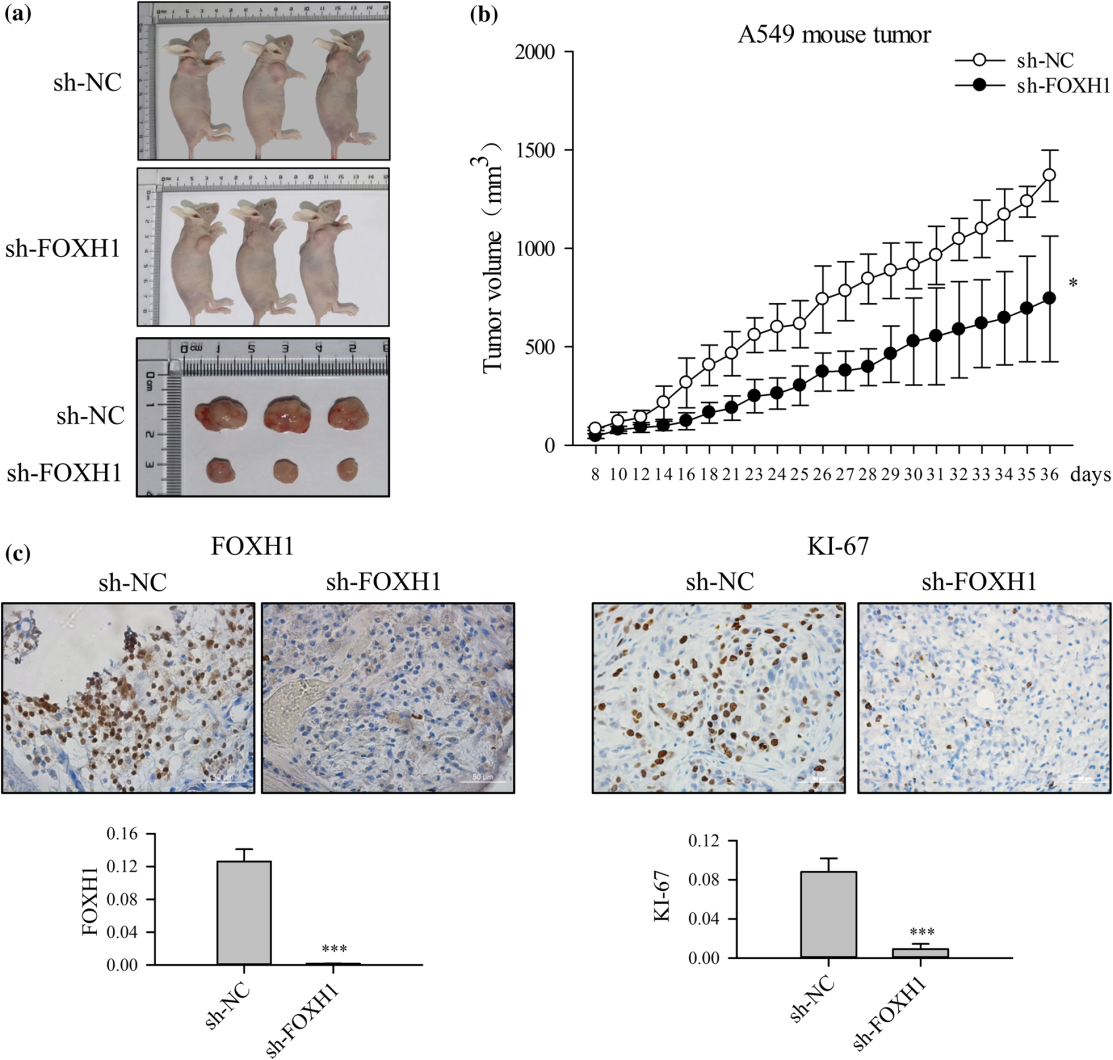
FOXH1 knockdown inhibits xenograft tumor growth in nude mice. **a** Growth of the tumor masses in A549 cells which transduced with sh-NC or sh-FOXH1 were implanted into the shoulders of nude mice. **b** Tumor growth curve in nude mice. Tumor volume measurement was performed by digital caliper after tumor cells were injected subcutaneously into the shoulders of nude mice for 8 days. When measurement was terminated, the animals were sacrificed on day 36, and tumor tissues were collected. FOXH1 knockdown cell group grew slower than the control group and formed tumor masses (*p < 0.05 vs. sh-NC). **c** Immunohistochemistry assay of FOXH1 and KI-67 expression from tumor xenografts in each group of nude mice. (***p< 0.001 vs. sh-NC)

To confirm the oncogenic effect of FOXH1 in vivo, a xenograft tumor-bearing model was established by inoculating sh-NC or sh-FOXH1 transfected A549 cells into the nude mice. Thirty-six days after inoculation, the xenografted tumors were harvested and the tumor sizes were measured. As shown in Fig. 3a, the tumors induced by sh-FOXH1 transfected A549 cells were significantly smaller than those induced by sh-NC transfected A549 cells in xenografted mice (Fig. 3a). Tumors induced by the sh-FOXH1 transfected cells grew significantly slower compared with those in the control mice (Fig. 3b). IHC analysis of tumor tissue verified that FOXH1 was barely expressed in the tumor induced by sh-FOXH1transfected cells (Fig. 3c). Additionally, we also examined the expression of Ki-67 antigen, a cellular proliferation marker in mouse tumors. As shown in Fig. 3c, the proportion of the Ki-67 positive cells was significantly decreased in the tumor induced by FOXH1 depleted A549 cells. Collectively, these results suggested that FOXH1 depletion efficiently suppresses tumor growth in vivo.

### Knockdown of FOXH1 suppresses lung cancer cell invasion and migration

Since cancer cell invasion and migration are essential for cancer advancement and malignancy, we investigated how FOXH1 expression affects the mobility of lung cancer cells.

**Fig. 4:**
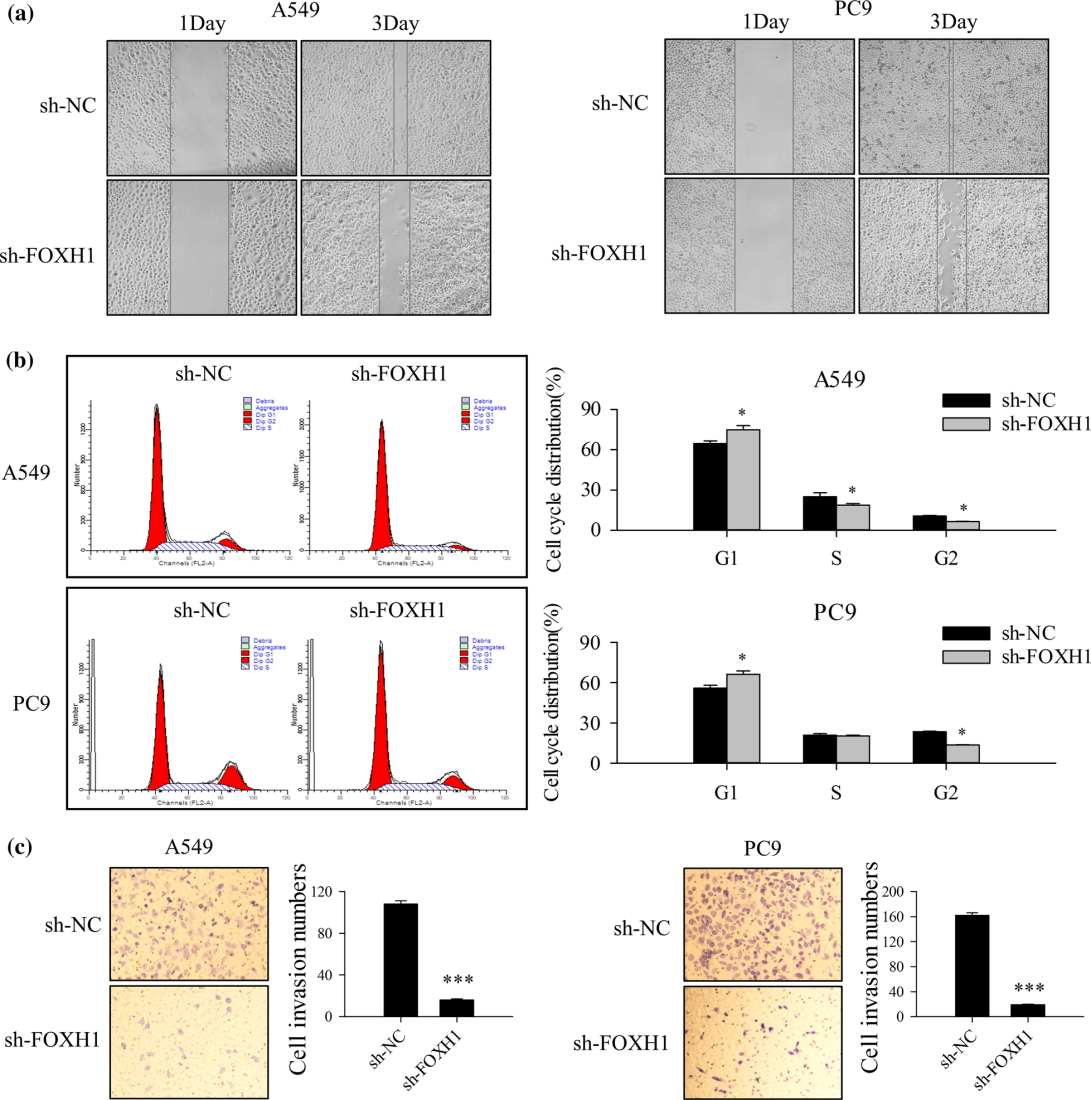
The effects of sh-FOXH1 on cell invasion, proliferation and migration of lung cancer cells. **a** Wound healing assay was performed to determine the migration ability of sh-FOXH1 transfected A549 and PC9 cells, the wounded area was examined by phase-contrast microscopy at 1 day and 3 days. **b** Cell cycle analyses in the sh-FOXH1 transfected A549 and PC9 cells by flow cytometry. (*p<  0.05 vs. sh-NC). **c** Transwell invasion assays was performed to determine cell invasion in sh-FOXH1 transfected A549 and PC9 cells. The invasion cells number were counted (***p<  0.001 vs. sh-NC)

Effects of FOXH1 on cell proliferation and migration were investigated through wound healing assay. FOXH1-suppressed A549 and PC9 cells showed a marked decrease proliferative and migration ability compared to the sh-NC control (Fig. 4a). Cell cycle progression was further assessed via flow cytometry. Knockdown of FOXH1 led to an increase in G1 phase from 64.5% to 74.88% (A549 cells) or 55.85% to 66.18% (*p* < 0.05) (PC9 cells), and decrease in G2 phase in A549 and PC9 cells from 10.52% to 6.40% or 23.30% to 13.55% (*p* < 0.05), and decrease in S phase in A549 and PC9 cells from 24.89% to 18.73% or 20.85% to 20.28% (Fig. 4b) indicating the FOXH1 knockdown inhibits cell cycle progression. In the transwell invasion assay, suppression of FOXH1 led to cell invasion decrease from 108 to 16 (*p* < 0.001) in A549 cells and from 162 to 19 (*p* < 0.001) in PC9 cells, relative to corresponding control groups (Fig. 4c). These results clearly suggest that knockdown of FOXH1 inhibits migration, proliferation and invasion of lung cancer cells.

### Knockdown of FOXH1 decreased EMT marker alterations in PC9 and A549 cells

As cellular invasion and migration are the main phenotypes of epithelial-mesenchymal transition (EMT), a process that involves in tumor metastatic expansion or cancer advancement, we further assessed whether FOXH1 knockdown affects EMT-specific molecules in lung cancer cells using the A549 and PC9 cell lines that stably express sh-FOXH1 and sh-NC.

**Fig. 5:**
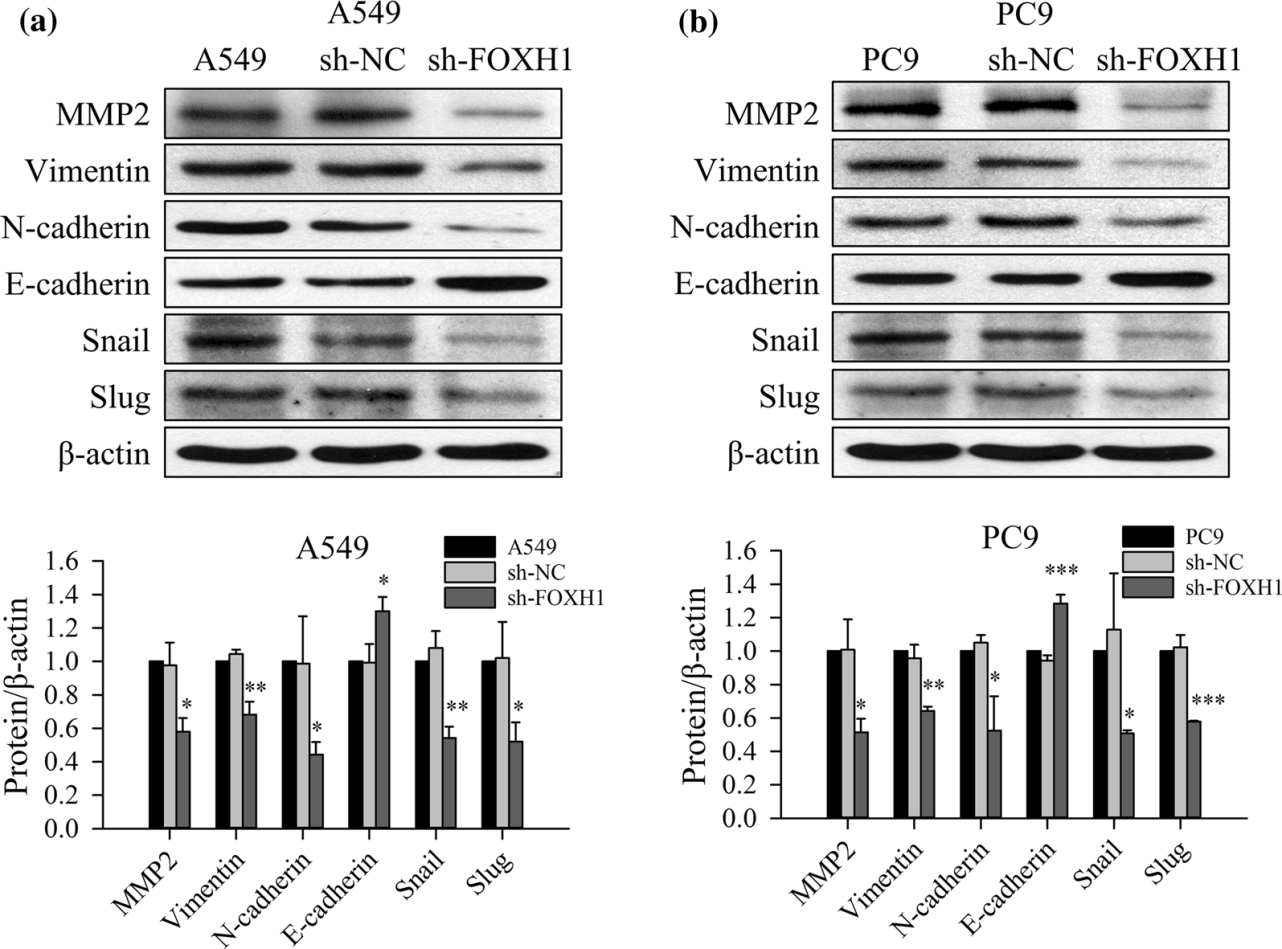
Knockdown of FOXH1 decreased EMT marker alterations in A549 and PC9 cells. **a** Western blot of EMT-related molecular markers, include MMP2, Vimentin, N-cadherin, E-cadherin, Snail and Slug in the sh-NC or sh-FOXH1 transfected A549 cells. β-actin is served as the internal control (*p< 0.05, **p < 0.01 vs. sh-NC). **b** Western blot of EMT-related molecular markers, include MMP2, Vimentin, N-cadherin, E-cadherin, Snail and Slug in the sh-NC or sh-FOXH1 transfected PC9 cells. β-actin is served as the internal control. Densitometry analyses were performed using ImageJ software and normalized to loading WB controls. (*p< 0.05, **p< 0.01, ***p< 0.001 vs. sh-NC)

Western blot analysis results of A549 and PC9 cells with FOXH1 knockdown are shown in Fig. 5. The mesenchymal markers Matrix metalloproteinase-2 (MMP2), Vimentin, N-cadherin, Snail and Slug were significantly down-regulated, whereas the epithelial markers E-cadherin was notably upregulated by FOXH1 depletion in both lung cancer cell lines (Fig. 5a, b). No changes were found in the negative control cell lines. Western blot results showed a decreased level of MMP2. Therefore, we performed gelatin zymography, which is a functional assay, to detect gelatinase in the culture supernatant of PC9 and A549 cells transfected with sh-NC or sh-FOXH1. Analysis of the presence of gelatinases by zymography revealed high levels of MMP2 in the supernatants of both sh-NC cell cultures of PC9 and A549, whereas knockdown of FOXH1 demonstrated a significant reduction of MMP2 activity (Additional file 3: Fig. S1). Our findings indicate that FOXH1 can regulate the metastatic potential of lung cancer cells via activation of EMT.

### Knockdown of FOXH1 suppresses β-Catenin signaling in lung cancer

Wnt/β-catenin signaling pathway is essential in regulating multiple processes, including cell proliferation, migration, and invasion in various cancer types, it may also play an important role in EMT. To further explore and clarify the molecular mechanisms underlying FOXH1-mediated tumor promotion and malignancy in lung cancer, effects of FOXH1 on Wnt/β-catenin signaling pathway were assessed. First, western blot was used to evaluate the levels of Wnt/β-catenin signaling molecules, namely β-catenin, cyclin D1 and p-GSK-3β.

**Fig. 6:**
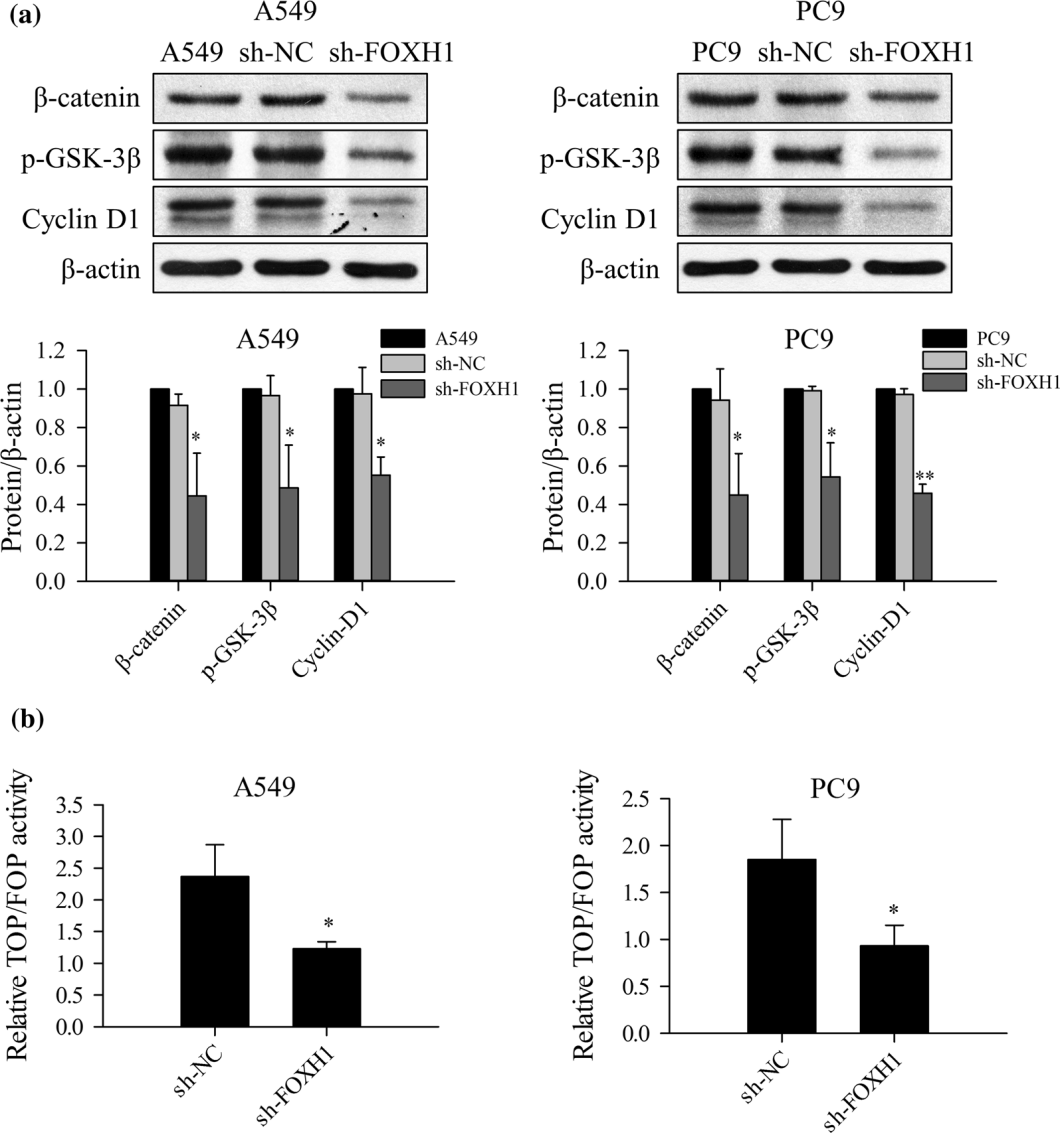
Knockdown of FOXH1 suppresses β-Catenin signaling in lung cancer. **a** Western blotting showing that FOXH1 knockdown decreased the expression of β-catenin, p-GSK-3β and cyclin D1 in A549 and PC9 cells. β-actin served as the internal control. Densitometry analyses were performed using ImageJ software and normalized to loading WB controls (***p< 0.001 vs. sh-NC). **b** TCF/LEF-dependent transcriptional activity of β-catenin in A549 and PC9 cells transfected with TOP/FOP flash reporter plasmids. Assays of relative luciferase activity in cells were performed (*p < 0.05 vs. sh-NC)

As shown in Fig. 6a, the protein levels of β-catenin and its two downstream targets, cyclin D1 and p-GSK-3β, were significantly decreased in A549 and PC9 cells transfected with sh-FOXH1. Next, the DLR assay further detected changes in β-catenin activity induced by FOXH1. TCF/LEF-dependent transcriptional activity of β-catenin in A549 and PC9 cells was measured after transfection with TOP/FOP flash reporter plasmids. Compare to the sh-NC control group, sh-FOXH1 induced a significant decrease in TOP luciferase reporter activity (Fig. 6b), thus a decreased Wnt/β-catenin activity. Based on the results above, we conclude FOXH1 exerts its activity upstream of the β-catenin, and consequently, its silencing inhibits activation of Wnt/β-catenin signaling in lung cancer.

## Discussion

Recently, a number of studies have reported the role of FOXH1 in various cancers. However, the detailed biological functions of FOXH1 in lung cancer and the underlying mechanisms have not yet been investigated. Analyses of data from the comprehensive public cancer database TCGA revealed higher expression level of FOXH1 in lung cancer tissues than in normal tissues. Of note, the high expression level of FOXH1 was closely related to poor patient prognosis. In addition, we observed that knockdown of FOXH1 expression significantly suppressed lung cancer cell proliferation, migration, and invasion. These results suggested that FOXH1 is involved in the progression of lung cancer.

EMT drives metastasis in the progression of multiple cancer types, including lung cancer [20, 21]. EMT is stimulated under conditions where epithelial cells lose connexins, such as E-cadherin, and acquire mesenchymal markers, such N-cadherin and vimentin [16]. MMPs are essential agents responsible for extracellular matrix degradation, and their abnormal expression is linked to the progression of many cancers. We performed gelatin zymography, a functional assay used to detect gelatinases in culture supernatants of lung cancer cells [18, 19]. Identification of EMT status may thus aid in clarifying the mechanisms underlying lung cancer metastasis. An association of FOXH1 with EMT has previously been reported [12, 13]. Here, we found that knockdown of FOXH1 significantly decreased the expression of mesenchymal markers (Slug, Snail, MMP2, N-cadherin, and Vimentin), while increasing the expression of epithelial marker (E-cadherin) in lung cancer cells. A mouse xenograft model demonstrated that knocking down FOXH1 inhibited tumor growth in vivo. Furthermore, knockdown of FOXH1 decreased the expression and secretion of MMP2, a key MMP associated with tumor dissemination and invasiveness. These results further support the involvement of FOXH1 in EMT induced progression in lung cancer.

β-catenin can be localized in the membrane, cytoplasm and nucleus of cells [22]. This protein exerts a dual role depending on its intracellular localization. When expressed on the membrane, β-catenin binds tightly to classical cadherins, regulates cell-to-cell adhesion and cell growth, and has a negative effect on tumor growth [23]. Cytoplasmic and nuclear β-catenin mainly act as central molecules in the Wnt signaling pathway [24]. The Wnt/β-catenin signaling pathway regulates cell morphogenesis, gene transcription, differentiation, and proliferation [5, 6]. Slug, Vimentin, MMPs, and other downstream target genes in the Wnt/β-catenin pathway are key regulators of EMT [7]. In the early stages of metastasis, cancer cells undergo an EMT-like process, which is controlled by Wnt/β-catenin signaling activation [8]. Aberrant Wnt/β-catenin signaling is involved in the progression of different tumor types through modulation of downstream targets, such as c-myc, cyclin D1, and GSK-3β [25–27]. We found that cyclin D1, β-catenin and GSK-3β levels were decreased in FOXH1 knockdown cells. To further verify the relationship between FOXH1 and β-catenin, we subsequently explored the signaling pathway involving these factors using the TOP/FOP luciferase reporter system. FOXH1 knockdown induced significant reduction of TOP/FOP luciferase activity, confirming that the Wnt/β-catenin pathway is a downstream target activated by FOXH1 in lung cancer cells.

The Wnt/β-catenin pathway has been proved to be crucial for various cancers. Therapeutic strategies targeting this signaling pathway are increasingly being proposed for cancer treatment. Although no drugs that specifically inhibit this signaling pathway have been approved yet, considerable efforts have been made towards their development. Investigating the mechanism and targets of the Wnt/β-catenin signaling pathway plays a limited role in facilitating the development of novel small-molecule inhibitors. Therefore, further exploration and evaluation are warranted to identify safe targeted agents and achieve optimal use with clinical benefits in cancer. Here, we found that the expression of FOXH1 was upregulated in lung cancer cells, and it was associated with a poor prognosis. FOXH1 promoted lung cancer progression and metastasis by activating the EMT process and Wnt/β-catenin signaling pathway. These findings provide new perspectives for the treatment of Wnt/β-catenin signaling pathway-dependent cancers.

## Conclusions

We demonstrated that FOXH1 activated Wnt/β-catenin signaling, which facilitated the EMT process in lung cancer cells. Suppressing FOXH1 expression could effectively inhibit lung cancer cell growth. These findings support the potential clinical utility of FOXH1 as a novel therapeutic target in lung cancer treatment.

## Supplementary Information

**Additional file 1: Table S1.** Antibody list.

**Additional file 2: Table S2. ** Components of MMP Zymography Analysis Kit (XF-P17750).

**Additional file 3:** Editing Certificate.

## Data Availability

The datasets used and/or analyzed during the current study are available from the corresponding author upon reasonable request.

## Abbreviations

CCK-8: Cell Counting Kit-8
DAB: Diaminobenzidine
DLR: Dual-Luciferase Reporter
DMEM: Dulbecco's modified Eagle's medium
EMT: Epithelial-mesenchymal transition
FBS: Fetal bovine serum
FOXH1: Forkhead box protein H1
IHC: Immunohistochemistry
MMP2: Matrix metalloproteinase
NC: Negative control
PBS: Phosphate-buffered saline
shRNA: Short hairpin RNA
TCGA: The Cancer Genome Atlas
