# Supplementary material for: FOXH1 promotes lung cancer progression by activating the Wnt/β-catenin signaling pathway
Source: Cancer Cell Int. 2021 Jun 5;21:293. doi: 10.1186/s12935-021-01995-9 (PMC8180118; doi:10.1186/s12935-021-01995-9)
Supplement: Supplementary file 2 — Additional file 2: Table S2. Components of MMP Zymography Analysis Kit (XF-P17750). [file 12935_2021_1995_MOESM2_ESM.docx]

Additional file 2 Table. Components of MMP Zymography Analysis Kit (XF-P17750)

| Components | Specification |
| --- | --- |
| 2×SDS-PAGE non-reducing buffer | 1.5ml |
| 10×Substrate G | 50ml |
| 10×Buffer A | 50ml |
| 10×Buffer B | 50ml |
| SDS-PAGE gel Coomassie Brilliant Blue staining | 50ml |
