# Supplementary figures and images for: FOXH1 promotes lung cancer progression by activating the Wnt/β-catenin signaling pathway
Source: Cancer Cell Int. 2021 Jun 5;21:293. doi: 10.1186/s12935-021-01995-9 (PMC8180118; doi:10.1186/s12935-021-01995-9)

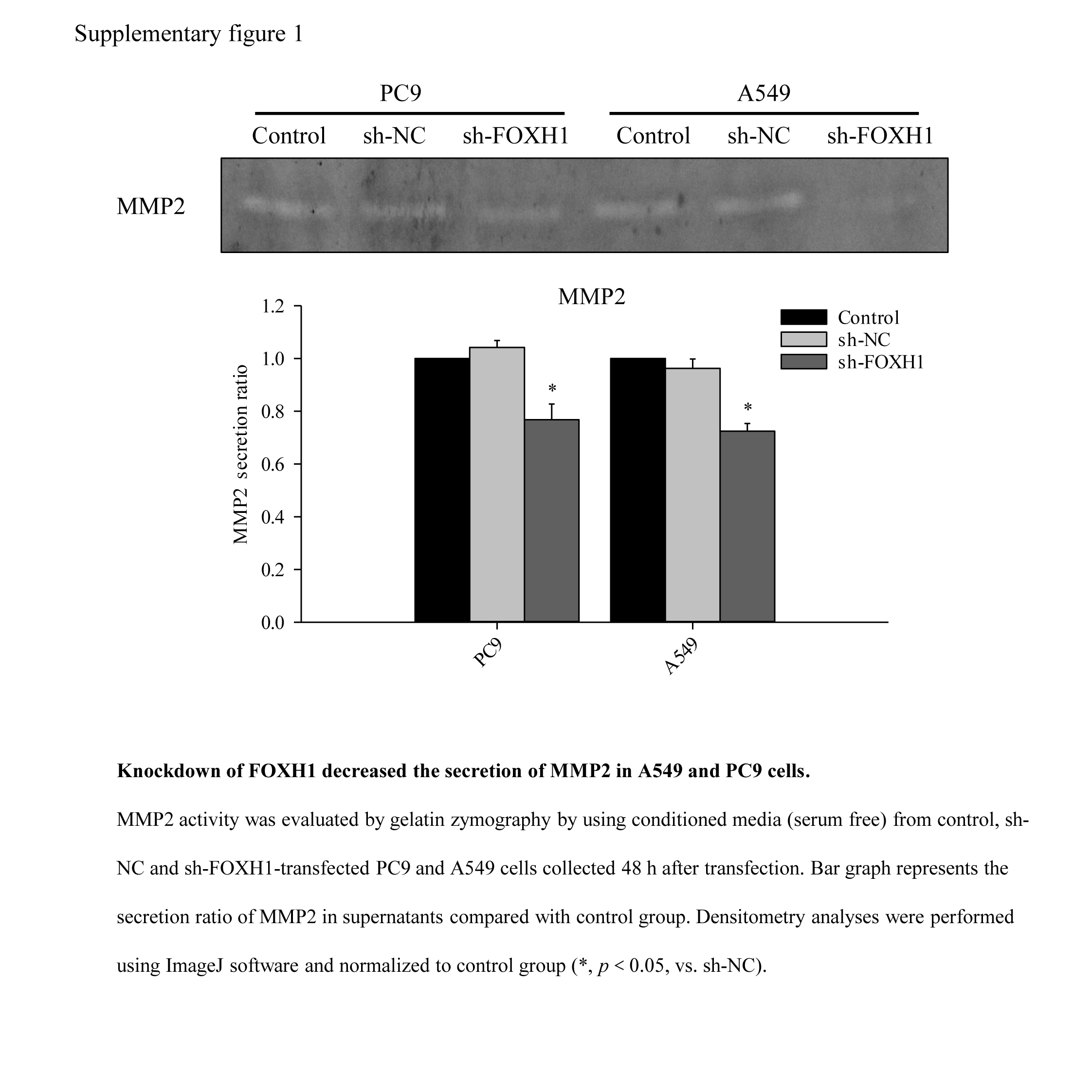

Supplement: Supplementary file 3 — Additional file 3: Editing Certificate. [file 12935_2021_1995_MOESM3_ESM.tif]
